# Unterschiede der Verletzungsmuster bei Motorradunfällen von Kindern und Jugendlichen

**DOI:** 10.1007/s00113-021-01090-8

**Published:** 2021-10-15

**Authors:** L. Oezel, C. Jaekel, D. Bieler, D. Stuewe, A. Neubert, R. Lefering, J. P. Grassmann, J. Windolf, S. Thelen, Sektion Notfall‑, Intensivmedizin und Schwerverletztenversorgung (Sektion NIS) der DGU

**Affiliations:** 1 Klinik für Orthopädie und Unfallchirurgie, Medizinische Fakultät und Universitätsklinikum Düsseldorf, Heinrich-Heine-Universität Düsseldorf, Moorenstraße 5, 40225 Düsseldorf, Deutschland; 2 Klinik für Unfallchirurgie und Orthopädie, Wiederherstellungs‑, Hand- und Plastische Chirurgie, Verbrennungsmedizin, Bundeswehrzentralkrankenhaus Koblenz, Koblenz, Deutschland; 3 Institut für Forschung in der Operativen Medizin, Universität Witten/Herdecke, Köln, Deutschland

**Keywords:** Motorrad, Mortalität, Verkehrsunfall, Verletzungen, TraumaRegister, Motorcycle, Mortality, Traffic accident, Injury, TraumaRegistry

## Abstract

**Hintergrund:**

Verkehrsunfälle und ihre Verletzungsfolgen stellen eine häufige traumatische Ursache für das Versterben und für das Auftreten von irreversiblen Schäden bei Kindern und Jugendlichen dar. Bei Motorradunfällen unterscheiden sich dabei Verletzungsmuster abhängig vom Patientenalter.

**Ziel der Arbeit:**

Ziel dieser Studie ist es, die typischen Verletzungsmuster nach Motorradunfällen im Kindes- und Jugendalter vergleichend zu beschreiben, da diese einen ausschlaggebenden Einfluss auf die Prävention sowie die adäquate Behandlung der jeweiligen Patientengruppen bieten können.

**Material und Methoden:**

In die Studie wurden innerhalb der Jahre 2002–2018 22.923 Patienten aus dem TraumaRegister der Deutschen Gesellschaft für Unfallchirurgie (DGU®) eingeschlossen. Die Auswertung erfolgte in 4 Altersgruppen: Gruppe 1 (4 bis 15 Jahre), Gruppe 2 (16 bis 17 Jahre), Gruppe 3 (18 bis 20 Jahre) sowie Gruppe 4 (21 bis 50 Jahre) als Kontrolle.

**Ergebnisse:**

In Gruppe 2 stellten Extremitätenverletzungen das häufigste Verletzungsmuster dar und traten überwiegend im Bereich der unteren Extremität auf. Zudem ergab die Analyse, dass Gruppe 1 häufiger ein schweres Schädel-Hirn-Trauma erleidet, trotz initial schlechtem Zustand aber ein besseres Outcome aufweist. Thorakale, abdominelle sowie Wirbelsäulen- und Beckenverletzungen kommen bei den jüngeren Altersgruppen seltener vor. Insbesondere stellen Rippenfrakturen eine Rarität bei Kindern dar. In der Diagnostik werden Kinder im Vergleich zu Erwachsenen seltener einer Ganzkörper-CT-Diagnostik zugeführt.

**Diskussion:**

Die Studie deckt altersabhängige Unterschiede in den Verletzungsmustern von Patienten auf, die als Beifahrer oder Fahrer eines Motorrades in einen Unfall involviert waren. Zudem konnten ebenso Unterschiede in der prä- und innerklinischen Versorgung herausgearbeitet werden.

## Abstract (en)

**Background:**

Traffic accidents and the traumatic injury consequences are frequent causes of mortality and irreversible damage in children and young adults. In motorcycle accidents the injury patterns differ depending on the age of the patient.

**Objective:**

The aim of this study was to describe the typical injury patterns after motorcycle accidents involving children and adolescents as these can have a decisive influence on the prevention and the adequate treatment in the respective patient groups.

**Material and methods:**

The study included 22,923 patients from the years 2002–2018 which were extracted from the TraumaRegister DGU®. Injury patterns of 4 age categories were analyzed: group 1 (4–15 years), group 2 (16–17 years), group 3 (18–20 years) and group 4 (21–50 years).

**Results:**

In both younger age groups, limb injuries mostly of the lower extremities, showed the highest incidence. Moreover, younger patients with traumatic brain injury showed better outcomes despite of initially poor conditions. Ribcage, abdominal, pelvic and spinal injuries are the least frequent in younger patients. In terms of diagnostics, children are less likely to undergo whole-body computed tomography (CT) diagnostics than adults.

**Conclusion:**

The study revealed age-specific differences with respect to injury patterns in patients involved in motorcycle accidents, either as drivers or co-drivers. Furthermore, the analysis of preclinical and in-hospital treatment elucidated the relevance of preventive and protective measures.

## Hintergrund

Verletzungen und Unfallfolgen sind für einen Großteil der Morbidität und Mortalität in Industrie- und Entwicklungsländern verantwortlich [28]. Verletzungen durch Verkehrsunfälle sind der häufigste Grund für das Auftreten von Behinderungen und einer der Hauptgründe für Mortalität bei Kindern und Jugendlichen < 18 Jahren [22, 23] – ab dem 5. Lebensjahr sogar die häufigste Todesursache [12]. Die Weltgesundheitsorganisation konnte Verkehrsunfälle als zehnthäufigste Todesursache weltweit identifizieren und prognostiziert einen Anstieg um bis zu 40 % bis zum Jahr 2030 [35]. Durch erhebliche Fortschritte in Technologie und Sicherheit in der Fahrzeugindustrie, konnte in den letzten Jahren v. a. in Industrieländern eine deutliche Reduktion von Verkehrsunfällen und assoziierter Unfallfolgen erzielt werden [33]. Trotz dieser Entwicklungen gibt es nach wie vor eine relevante Anzahl von Verkehrsunfällen, die zu schweren Verletzungen und Todesfällen führen. Motorradfahrer stellen hierbei die am stärksten gefährdete Gruppe der Verkehrsteilnehmer dar und haben im Vergleich zu Insassen eines Autos ein über 30-fach erhöhtes Risiko, bei einem Verkehrsunfall zu versterben [25]. Morbidität und Mortalität infolge von Motorradunfällen werden in vielen Ländern sogar als öffentliches Gesundheitsproblem diskutiert [34]. In früheren Studien konnte gezeigt werden, dass die gezielte Forschung und Analyse dieser Thematik einen großen Einfluss auf die Prävention von Motorradunfällen und die Optimierung medizinischer Behandlungen haben kann [8]. Viele Arbeiten hinsichtlich dieser Thematik zielen auf die Untersuchung epidemiologischer Aspekte sowie auf die Häufigkeitsverteilung von Verletzungsmustern erwachsener Motorradfahrer ab. Bachulis et al. beschrieben in ihrer Studie Verletzungen am Bewegungsapparat, einschließlich Frakturen, als häufigste Unfallfolge [5]. Korrespondierend hierzu demonstrierten Kortor et al., dass Frakturen und knöcherne Läsionen im Bereich der unteren Extremität die häufigste Verletzung unter Motorradfahrern darstellen [21]. In den letzten Jahren gewannen Motorradunfälle, in welche Kinder und Jugendliche involviert waren und verletzt wurden, zunehmend an Aufmerksamkeit, da Kinder als Beifahrer auf Motorrädern die weitestgehend verletzlichste Population darstellen [6, 20, 29].

In der vorliegenden Studie werden die typischen Verletzungsmuster bei Kindern und Jugendlichen innerhalb der Altersstufen 4 bis 15 Jahre, 16 bis 17 Jahre sowie 18 und 20 Jahre (primäre Zielgruppen) untersucht und mit Verletzungsmustern Erwachsener innerhalb der Altersstufe 21 bis 50 Jahre (Kontrollgruppe) nach Motoradunfällen verglichen. Des Weiteren werden die Mortalität und die Krankenhausliegedauer untersucht. Hieraus sollen relevante Unterschiede aufgedeckt werden, die zur Optimierung von klinischen Abläufen sowie für präventive Zwecke genutzt werden können.

## Material und Methoden

Das TraumaRegister der Deutschen Gesellschaft für Unfallchirurgie (DGU®) wurde 1993 gegründet. Ziel dieser multizentrischen Datenbank ist eine pseudonymisierte und standardisierte Dokumentation von Schwerverletzten.

Die Daten werden prospektiv in 4 aufeinanderfolgenden Phasen gesammelt: A) prähospitale Phase, B) Schockraum und anschließende OP-Phase, C) Intensivstation und D) Entlassung. Die Dokumentation beinhaltet detaillierte Informationen über Demographie, Verletzungsmuster, Komorbiditäten, prähospitales und innerklinisches Management, intensivmedizinischen Verlauf, wichtige Laborbefunde einschließlich Transfusionsdaten, sowie das Patientenoutcome. Hierbei kann das Outcome der in das Traumaregister aufgenommenen Patienten nur bis zur Entlassung aus dem Krankenhaus untersucht werden. Das Einschlusskriterium für das TraumaRegister DGU® ist die unfallbedingte Aufnahme in das Krankenhaus über den Schockraum mit anschließender Intensiv- oder IMC-Überwachung oder die Ankunft in der Klinik mit Vitalzeichen und Versterben vor Aufnahme auf die Intensivstation. Die Infrastruktur für Dokumentation, Datenmanagement und Datenanalyse wird von der AUC – Akademie der Unfallchirurgie GmbH, welche der DGU angegliedert ist, bereitgestellt. Die wissenschaftliche Führung liegt bei der Sektion Notfall‑, Intensivmedizin und Schwerverletztenversorgung der DGU (Sektion NIS). Über eine webbasierte Anwendung geben die teilnehmenden Kliniken ihre pseudonymisierten Daten in eine zentrale Datenbank ein. Wissenschaftliche Auswertungen werden nach einem Reviewverfahren der Sektion NIS genehmigt [13].

Die teilnehmenden Kliniken sind primär in Deutschland (90 %) lokalisiert, aber eine zunehmende Anzahl von Kliniken aus anderen Ländern steuert ebenfalls Daten bei (zurzeit Österreich, Belgien, Finnland, Luxemburg, Slowenien, Schweiz, Niederlande und die Vereinigten Arabischen Emiraten). Derzeit fließen jährlich knapp 30.000 Fälle (Basiskollektiv) von über 650 Kliniken in die Datenbank ein. Die Teilnahme am TraumaRegister DGU® ist für die dem TraumaNetzwerk DGU® zugehörigen Kliniken verpflichtend.

Die vorliegende Arbeit steht in Übereinstimmung mit der Publikationsrichtlinie des TraumaRegister DGU® und ist unter folgender Projektnummer registriert: TR-DGU-Projekt-ID 2020-007.

### Patienten

Die analysierten Daten entstammen dem Standarderhebungsbogen der teilnehmenden deutschsprachigen Kliniken (Deutschland, Österreich, Schweiz) und umfassen den Zeitraum von 2002 bis 2018. Eingeschlossen wurde das sog. Basiskollektiv des TraumaRegister DGU®, d. h., Patienten mit einer Maximum Abbreviated Injury Scale (MAIS) = 2, die entweder verstorben sind oder auf der Intensivstation versorgt wurden, sowie alle Patienten mit einer MAIS ≥ 3. Ausschlusskriterien waren ein Alter < 4 und > 50 Jahre. Für die Auswertung wurde folgende Kategorisierung des Alters vorgenommen: Gruppe 1 (4 bis 15 Jahre), Gruppe 2 (16 bis 17) Jahre sowie Gruppe 3 (18 bis 20 Jahre). Diese 3 Gruppen werden zusammenfassend als Zielgruppe bezeichnet; Gruppe 4 (21 bis 50 Jahre) wird als Kontrollgruppe betrachtet. Die Gruppeneinteilung erfolgte im Hinblick auf die Art der Teilnahme am Straßenverkehr. Dabei sind die eingeschlossenen Patienten der Gruppe 1 als Beifahrer zu bewerten, die Gruppe 2 als Nutzer eines Kleinkraftrads (Moped), Gruppe 3 als Fahranfänger eines Motorrads und Gruppe 4 als Vergleichsgruppe. Die Art des Motorrads bzw. die Position (Fahrer/Beifahrer) wird im TR-DGU nicht erfasst. Ebenso wird der Konsum von Alkohol (erst ab der Datensatz-Revision 2015 aufgenommen und kein Pflichtfeld) und anderen Drogen nicht berücksichtigt.

Vergleichend werden folgende Parameter dargestellt: Verletzungsmuster, präklinische und klinische Versorgung, Outcome (Liegedauer, Mortalität).

### Statistik

Die statistische Auswertung erfolgte deskriptiv mittels SPSS (Version 24, IBM Inc., Armonk, NY, USA). Die Darstellung erfolgt mit Fallzahl und Prozenten bzw. Mittelwerten und Standardabweichung (SD). Bei nicht normal verteilten Daten wurde der Median mit „interquartil range“ (IQR) angegeben. Wegen der großen Fallzahl und der Vielzahl möglicher Vergleiche (multiple Parameter im 4‑Gruppen-Vergleich) wurden keine formalen statistischen Tests durchgeführt.

## Ergebnisse

In die Studie wurden insgesamt 22.923 Patienten aus 660 deutschsprachigen Kliniken eingeschlossen. Die jeweilige Gruppenstärke betrug für Gruppe 1 (*n* = 508), Gruppe 2 (*n* = 2437), Gruppe 3 (*n* = 2218) und damit für die Zielgruppen, *n* = 5163. Die Häufigkeiten der jeweiligen Altersverteilungen der 3 Zielgruppen werden in Tab. 1 veranschaulicht. Für die Gruppe 4, die als Kontrollgruppe diente, betrug die Gruppenstärke (*n* = 17.760). Das männliche Geschlecht war mit 89 % der Fälle mit einer Gesamtanzahl von 20.368 deutlich häufiger betroffen als das Weibliche.

**Table 1:** 

Alter (Jahre)	Häufigkeit *n* (%)
4	2 (0,0)
5	2 (0,0)
6	6 (0,1)
7	2 (0,0)
8	7 (0,1)
9	4 (0,1)
10	9 (0,2)
11	17 (0,3)
12	20 (0,4)
13	35 (0,7)
14	71 (1,4)
15	333 (6,4)
16	1149 (22,3)
17	1288 (24,9)
18	778 (15,1)
19	714 (13,8)
20	726 (14,1)
Gesamt	5136 (100)

**Figure 1:**
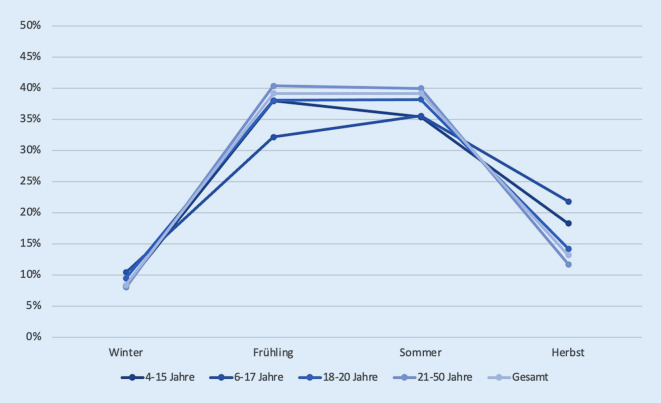

Hinsichtlich der Jahreszeiten konnte in allen 4 Gruppen die Mehrzahl der Patienten im Frühjahr sowie Sommer verzeichnet werden. Im Winter trat in allen Altersgruppen erwartungsgemäß die geringste Anzahl an Motorradunfällen auf (Abb. 1).

In allen 4 Gruppen wurde die Mehrzahl der Patienten primär in ein überregionales Traumazentrum (ÜTZ) verbracht (Gruppe 1: 66,3 %, Gruppe 2: 59,7 %, Gruppe 3: 67 %, Gruppe 4: 67,4 %). Die restliche Anzahl an Patienten wurde in ein regionales Traumazentrum (RTZ) oder lokales Traumazentrum (LTZ) verbracht (Tab. 2). Insgesamt wurden 93,9 % der 4‑ bis 15-Jährigen im primär angesteuerten Krankenhaus behandelt; nur 6,1 % dieser Patienten wurden zur Weiterbehandlung in ein anderes Krankenhaus verlegt. Auch die anderen Gruppen zeigten vergleichbare Werte für die Behandlung im primär angefahrenen Krankenhaus (Tab. 2).

**Table 2:** 

	4–15 Jahre (*n* = 508)		16–17 Jahre (*n* = 2437)		18–20 Jahre (*n* = 2218)		21–50 Jahre (*n* = 17.760)	
Allgemeine Daten								
Alter (MW/SD)	14,2	±2,0	16,53	±0,5	19,0	±0,9	35,6	±9,4
ISS (MW/SD)	16,4	±11,4	17,5	±12,8	18,5	±13,1	19,4	±12,8
Geschlecht, männlich (*n*/%)	406	80,1 %	2077	85,3 %	1997	90,2 %	15.888	89,7 %
Präklinik								
Prähospitale Zeit (MW/SD)	63,8	±25,9	61,7	±24,1	62,5	±28,4	61,5	±26,0
Bodentransport (*n*/%)	299	64,6 %	1480	67,8 %	1287	64,2 %	9979	62,4 %
Lufttransport (*n*/%)	164	35,4 %	702	32,2 %	717	35,8 %	6020	37,6 %
Primär versorgt (*n*/%)	477	93,9 %	2244	92,1 %	2061	92,9 %	16.509	93,0 %
Traumazentrum								
Überregionales Traumazentrum (*n*/%)	318	66,3 %	1419	59,7 %	1463	67,0 %	11.688	67,4 %
Regionales Traumazentrum (*n*/%)	124	25,8 %	745	31,4 %	567	26,0 %	4448	25,6 %
Lokales Traumazentrum (*n*/%)	38	7,9 %	211	8,9 %	152	7,0 %	1208	7,0 %
Klinischer Verlauf								
Ganzkörper-CT erfolgt (*n*/%)	350	69,4 %	1798	74,8 %	1749	79,6 %	14.115	80,2 %
Zeit bis Ganzkörper-CT (Median/IQR)	18	(13–27)	18	(13–26)	18	(13–26)	19	(13–27)
Anzahl der Diagnosen (Median/IQR)	4	(2–5)	4	(3–6)	4	(3–6)	5	(3–7)
Verletzte Körperregion AIS > 2 (Median/IQR)	2	(1–2)	2	(1–3)	2	(1–3)	2	(1–3)
ICU-Liegedauer (Tage, Median/IQR)	2	(1–5)	2	(1–5)	2	(1–6)	2	(1–8)
Krankenhausliegedauer (Tage, Median/IQR)	11	(5–19)	11	(6–19)	12	(6–21)	14	(8–25)
Verstorben (*n*/%)	23	4,5 %	126	5,2 %	116	5,2 %	930	5,2 %
Verstorben innerhalb 24 h (*n*/%)	16	3,1 %	92	3,8 %	81	3,7 %	634	3,6 %

*ISS* Injury Severity Score, *AIS* Abbreviated Injury Scale, *ICU* „intensiv care unit“, *MW* Mittelwert, *IQR* Interquartilsabstand

Die Analyse der Transportmittel ergibt, dass in allen Altersstufen der bodengebundene Transport dominiert. Es wurden 64,6 % der Patienten bodengebunden in das Traumazentrum transportiert und 35,4 % der Patienten luftgebunden transportiert (Tab. 2). Die Zeitspanne vom Transport der Patienten vom Unfallort in die Klinik weist für alle Altersstufen ähnliche Werte auf (Tab. 2).

Die Anzahl der durchgeführten Ganzkörper-CT-Untersuchungen war in Gruppe 1 (69,4 %) sowie Gruppe 2 (74,8 %) am geringsten. Innerhalb der Gruppen 3 und 4 wurden, insgesamt betrachtet, am häufigsten Ganzkörper-CT-Diagnostiken durchgeführt (Gruppe 3: 79,6 %, Gruppe 4: 80,3 %) (Tab. 2). Die Anzahl der Diagnosen steigt ebenso mit dem Alter der Patienten an (Gruppe 1: 4,1 (SD 2,3) Gruppe 2: 4,8 (SD 3,0), Gruppe 3: 4,9 (SD 2,9), Gruppe 4: 5,2 (SD 3,0)) (Tab. 2). Die Gesamtverletzungsschwere wurde mit dem Injury Severity Score (ISS) gemessen. Dabei steigt der ISS mit dem Alter der Patienten an (Tab. 2).

**Figure 2:**
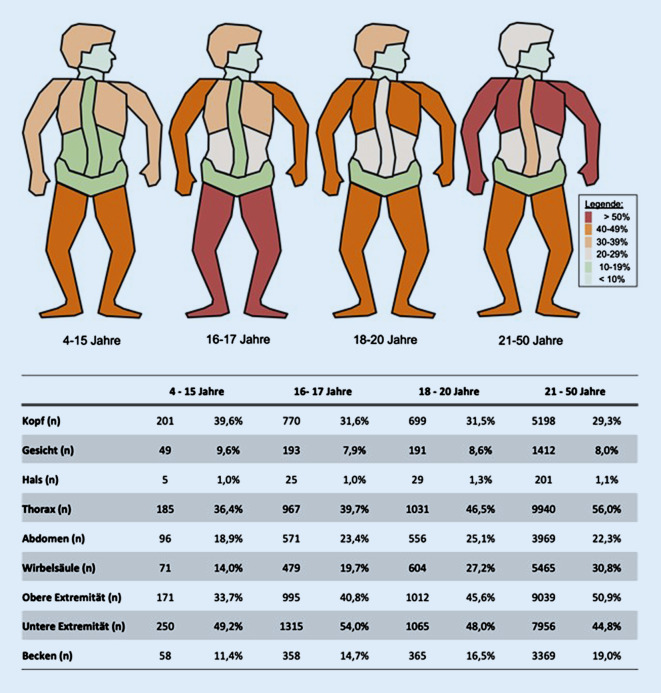

Hinsichtlich des Verletzungsmusters und der Verletzungsschwere, gemessen am Abbreviated Injury Scale (AIS), konnte bezüglich der Körperregionen und der Altersgruppen eine spezifische Verteilung festgestellt werden. Schädel-Hirn-Verletzungen traten bei Gruppe 1 am häufigsten auf (39,6 %). Die folgenden Altersstufen (Gruppe 2: 31,6 %, Gruppe 3: 31,5 %) wiesen ähnliche Werte wie Patienten des Vergleichskollektivs (Gruppe 4: 29,3 %) auf und waren um 8 bis 10 Prozentpunkte seltener betroffen. Sehr schwere Kopfverletzungen (AIS > 4) bestanden eher bei jüngeren Patienten mit sinkender Anzahl im Alter (Abb. 2). Schäden der Gesichts- und Halsregion traten insgesamt und gruppenübergreifend am seltensten auf (Abb. 2).

**Figure 3:**
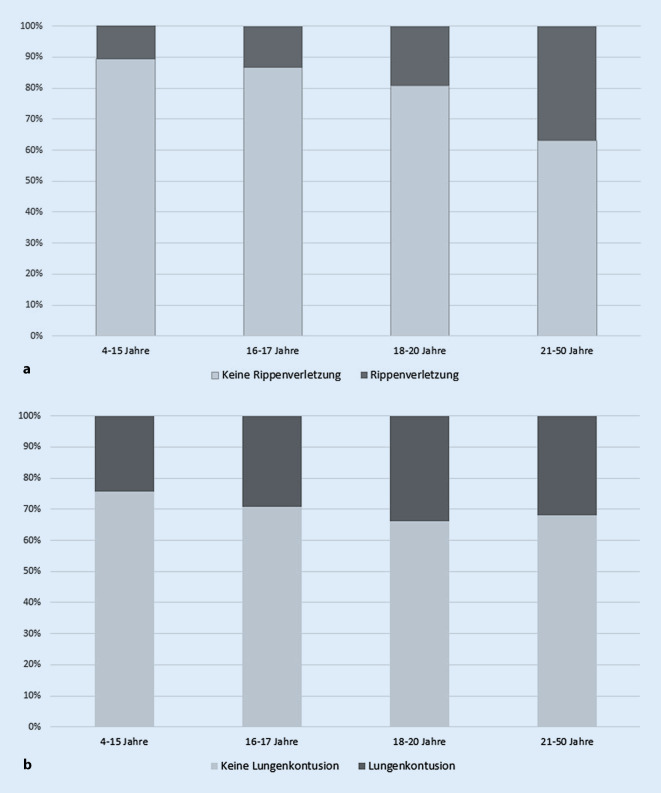

**Figure 4:**
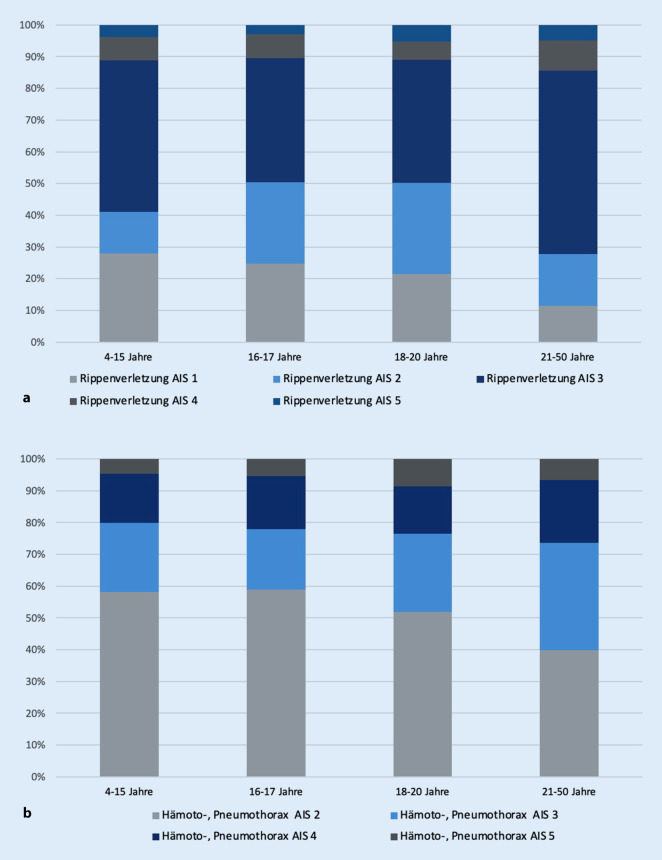

Thoraxverletzungen zeigten im Vergleich zu oben genannten Verletzungen eine häufigere Inzidenz, mit größerer Verletzungsanzahl in den Altersstufen der Gruppe 3 (46,5 %) und Gruppe 4 (56 %). In den jüngeren Patientengruppen konnte ein geringeres Aufkommen ermittelt werden (Gruppe 1: 36,4 %, Gruppe 2: 39,7 %). Eine detaillierte Darstellung der vorliegenden Verletzungen im Bereich des Thorax erbrachte hinsichtlich Rippenverletzungen insgesamt ein geringeres Vorkommen in den Gruppen 1, 2 und 3 im Vergleich zu Gruppe 4 (11 %, 13 %, 19 % vs. 37 %, Abb. 3a und 4a). Lungenkontusionen zeigten in Gruppe 3 (33,9 %) das häufigste Vorkommen (Gruppe 1: 24,2 %, Gruppe 2: 29,1 %, Gruppe 3: 33,9 %, Gruppe 4: 32 %, Abb. 3b). Pneumothoraces sowie Hämatopneumothoraces kamen in allen Gruppen zwischen 12,8 % und 25,4 % vor (Abb. 4b).

Abdominelle Verletzungen wiesen eine geringere Inzidenz mit dem höchsten Vorkommen in Gruppe 3 (25,1 %) auf (Abb. 2). Wirbelsäulenverletzungen nahmen mit dem Alter der Patienten zu und traten im Vergleich zwischen Gruppe 1 und Gruppe 4 doppelt so häufig bei den älteren Patienten auf (Abb. 2). Beckenverletzungen traten insgesamt selten auf, beginnend mit 11,4 % (Gruppe 1) und graduell ansteigenden Werten mit Anstieg des Patientenalters (Gruppe 4: 19,0 %) (Abb. 2).

Zusammengenommen, stellen Extremitäten entlang aller Altersklassen die am häufigsten betroffene Region dar, wobei die untere Extremität, außer in der Kontrollgruppe, häufiger verletzt ist als die obere Extremität. Im Bereich der oberen Extremität waren lediglich 33,7 % der Patienten aus Gruppe 1 betroffen. Innerhalb der Gruppe 2 waren es 40,8 %, innerhalb der Gruppe 3 zeigten sich 45,6 % betroffen. Patienten aus dem Vergleichskollektiv der Gruppe 4 wiesen mit 50,9 % die höchste Anzahl an Verletzungen der oberen Extremität auf. Im Bereich der unteren Extremität zeigten sich die meisten Verletzungen in den beiden jüngeren Altersgruppen (Gruppe 1: 49,2 %, Gruppe 2: 54,0 %). Gruppen 3 und 4 wiesen im Vergleich etwas niedrigere Werte auf (Gruppe 3: 48 %, Gruppe 4: 44,8 %). Die Analyse der Verletzungen des Oberschenkels ergibt, dass die 3 jüngeren Altersgruppen insgesamt häufiger betroffen sind als Gruppe 4 (Abb. 2; Tab. 1).

**Figure 5:**
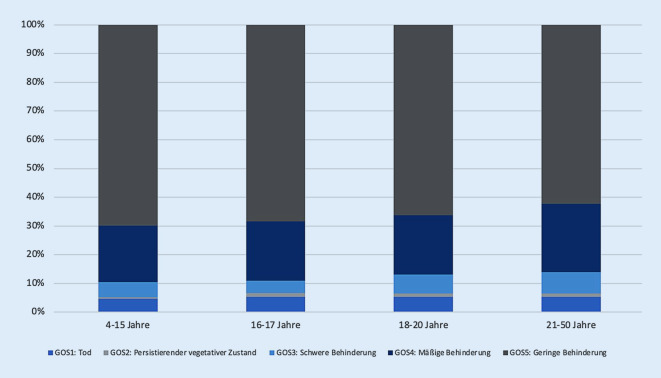

Das Outcome von Patienten mit Schädel-Hirn-Trauma wurde anhand der Glasgow Outcome Scale (GOS) bei Entlassung aus dem Primärkrankenhaus ausgewertet. Hierbei konnte innerhalb aller Altersgruppen bei 60–70 % der Patienten mit einem GOS-Wert von 5 ein positives Outcome mit allenfalls geringer Behinderung festgestellt werden. Etwa 20 % der Betroffenen aller Altersgruppen wiesen eine mäßige Behinderung (GOS = 4) auf. Des Weiteren hatten jeweils ca. 5–7 % der Verunfallten aller Altersstufen eine „schwere Behinderung“ (GOS = 3) bei Entlassung. Ein persistierend vegetativer Zustand (GOS = 2) stellte eine Seltenheit dar. Verstorben sind rund 5 % aller Patienten (jeweils Gruppen 1–4) (Abb. 5).

Bezüglich der Gesamtmortalität wurden in den Gruppen 2, 3 und 4 die gleichen Werte nachgewiesen (5,2 %). Die Gesamtmortalität in Gruppe 1 war mit 4,5 % diskret geringer. Das 95 %-Konfidenzintervall bot eine Unsicherheit von 2,7–6,3. Die Krankenhausliegedauer nahm mit den Altersgruppen zu. Dabei war der Aufenthalt auf der Intensivstation in allen Gruppen annähernd gleich (Tab. 2).

## Diskussion

Insgesamt wurden die spezifischen Verletzungsmuster von Kindern und Jugendlichen im Rahmen von Motorradunfällen bisher nur selten untersucht, und es gibt nur eine begrenzte Anzahl an Studien, die sich damit beschäftigt haben. Daniels et al. beschreiben eine hohe Inzidenz von Kopf- und Wirbelsäulenverletzungen bei Kindern, die als Beifahrer in einen Motorradunfall involviert waren [11]. Hier konnte zudem festgestellt werden, dass die Nutzung eines Schutzhelms zu einer signifikanten Reduktion schwerwiegender Kopfverletzungen beiträgt [26]. Die Studie von Hogue et al. untersuchte muskuloskeletale Verletzungsfolgen bei Kindern nach Motorradunfällen und demonstrierte eine signifikant erhöhte Verletzungsrate bei Kindern, die als Beifahrer eines Motorrads verunfallt sind, im Vergleich zu Kindern, die beispielsweise als Insassen eines Kraftfahrzeugs verunfallt sind. Die Kinder, die im Rahmen eines Motorradunfalls einen Unfall erlitten haben, zeigten dabei eine hohe Anzahl an Frakturen im Bereich der unteren Extremität (v. a. Tibiaschaftfrakturen) sowie Handgelenkfrakturen [19]. Miller et al. beschrieben ebenso pädiatrische Verletzungen im Rahmen von Motorradunfällen und konnten demonstrieren, dass Schutzvorrichtungen grundsätzlich vernachlässigt werden, jedoch die Nutzung dieser zu einer geringeren Verletzungsschwere beitragen [27]. Andere Studien beschäftigten sich mit kinematischen Aspekten von Motorradunfällen und benannten die schwerwiegendsten Mechanismen hinsichtlich Motorradunfällen [9]. Fan et al. beschrieben 2019, dass Vorschulalter, Sitzposition sowie höhere Geschwindigkeiten das Risiko von schweren Verletzungen bei Kindern als Beifahrer eines Motorrads erhöhen können. Zudem postulierte diese Studie, dass weitere Nachforschungen sowie gesetzliche Restriktionen hinsichtlich einer Altersbegrenzung, der Verwendung eines Kopfschutzes, einer adäquaten Sitzposition sowie eine Geschwindigkeitsbegrenzung zu einer deutlichen Reduktion von Verletzungsfolgen im Kindesalter führen würden [15].

Insgesamt stellten diese Studien fest, dass die Analyse dieser Thematik unter Berücksichtigung der Verletzungsmechanismen sowie der Verletzungsmuster und ihrer Häufigkeit zu einer deutlichen Optimierung in Versorgung und Prävention führen können.

Anhand dieser Studie konnte aufgezeigt werden, dass abdominelle Verletzungen, Wirbelsäulen- und Beckenverletzungen in den beiden jüngeren Altersgruppen insgesamt seltener als in der Vergleichsgruppe vorkamen. Im Gegensatz hierzu stellten Extremitätenverletzungen das häufigste Verletzungsmuster dar und traten überwiegend im Bereich der unteren Extremität auf. Dabei waren jugendliche Patienten deutlich häufiger betroffen als ältere Patienten. Dies unterstützt Ergebnisse von vorangegangenen Studien, die ebenso Verletzungen der unteren Extremität bei Jugendlichen als häufigste Verletzungsregion postulierten [21]. Dieser Aspekt lässt sich darauf zurückführen, dass Extremitäten im Vergleich zu anderen Körperregionen ungeschützte Bereiche darstellen, für die nur in geringerem Umfang präventive Maßnahmen umsetzbar erscheinen. Bisher ist in der Literatur wenig über die Schutzkleidung zur Prävention von Extremitätenverletzungen bei Motorradunfällen bekannt. Insbesondere gibt es hierzu keine Daten in Bezug auf Kinder und Jugendliche. Insgesamt konnte festgestellt werden, dass die Verwendung von Motorradschutzbekleidung bei Erwachsenen (Motorradjacken, Motorradhosen, Rumpfschutz, Handschuhe) mit einem niedrigeren Verletzungsrisiko, einer niedrigeren Verletzungsschwere und einer geringeren Hospitalisation nach Motorradunfällen assoziiert ist. Jedoch konnte keine Assoziation zu einem geringeren Frakturrisiko hergestellt werden [30–32]. Eine Ausnahme stellten kniehohe und sprunggelenkhohe Motorradstiefel dar, die zu einem signifikant niedrigeren Frakturrisiko im Bereich des Fußes und des Sprunggelenks führten [36]. Neben diesen Ergebnissen zeigte sich ein substanzieller Teil der Motorradschutzbekleidung nach einem Unfall stark beschädigt, sodass eine Weiterentwicklung und Verbesserung dieser Produkte empfohlen wurde.

Spezielle Schutzanzüge zur Protektion von Extremitäten stellen hier, v. a. in Bezug auf Kinder, mit Extremitätenverletzungen als häufigstem Verletzungsmuster, ein relevantes Thema dar, welches in zukünftigen Studien weiterverfolgt werden sollte.

Schwerwiegende Schädel-Hirn-Traumata treten am häufigsten in dem jüngsten Patientenkollektiv auf. Diese Patienten wiesen aber dennoch ein besseres Outcome auf, was auf ein verbessertes Regenerationspotenzial hinweist. Dabei bezieht sich die Beurteilung des Outcome auf den Zeitraum des Krankenhausaufenthalts. Korrespondierend hierzu konnten bereits andere Arbeitsgruppen eine hohe Rekonvaleszenz bei Kindern nach Kopfverletzungen feststellen [2, 3]. In diesem Zusammenhang wurde die Verwendung von ergänzenden Kopfschutzmaßnahmen (Helm ist bereits Pflicht) ausgiebig untersucht und konnte einen signifikanten Sicherheitsgewinn mit deutlicher Reduktion schwerwiegender Schädel- sowie Gesichtsverletzungsfolgen unterstreichen [24, 26].

Ebenso stellt sich die Frage, ob die präklinische Versorgung durch den Traumamechanismus oder den vorliegenden klinischen Befund beeinflusst wird. Hier zeigt sich bei der Untersuchung der Wahl des Transportmittels, dass die überwiegende Anzahl der Unfallverletzten, die als Beifahrer oder Fahrer eines Motorrads in einen Unfall verwickelt wurden, bereits initial in ein ÜTZ verbracht werden und der luftgebundene Transport, verglichen mit dem Gesamtkollektiv des TraumaRegister DGU®, bevorzugt wird [13]. Da Motorradunfälle häufiger außerhalb von Städten und Ortschaften stattfinden bedingen sich beide oben genannten Faktoren und bestätigen wiederum, dass hierdurch nur wenige dieser Patienten sekundär weiterverlegt werden mussten und somit bereits von Beginn an eine zielführende Therapie eingeleitet werden konnte.

Im Kontext der innerklinischen Versorgung lassen sich verschiedene Aspekte betrachten.

In Bezug auf die Verletzungsschwere ergibt sich hinsichtlich des ISS sowie der Anzahl der Verletzungsdiagnosen ein Anstieg mit dem Alter der Patienten. Folglich ist auch die Krankenhausliegedauer mit zunehmendem Alter länger. Dies wird auch in der Studie von Eden et al. belegt, wo Patienten, die älter als 65 Jahre waren und in Motorradunfälle verwickelt wurden, eine längere Verweildauer auf der Intensivstation sowie insgesamt im Krankenhaus aufwiesen [14]. Ebenso weist in dieser Studie die benannte Altersgruppe eine erhöhte Mortalität auf. In unserer Studie zeigt sich hinsichtlich des Sterblichkeitsrisikos innerklinisch kein wesentlicher Unterschied in den verschiedenen Altersklassen. Wie viele Patienten bereits vor Ort versterben, wird durch das TraumaRegister DGU® nicht erfasst.

Die Indikation zur Durchführung einer Ganzkörper-CT-Diagnostik wird mit zunehmendem Alter großzügiger gestellt. Dies bedeutet im Rückschluss, dass innerhalb unserer Studie in den Gruppen 1 und 2 insgesamt weniger Ganzkörper-CT-Untersuchungen durchgeführt wurden als in den Gruppen 3 und 4. Inwiefern dies zu einer Verzerrung des diagnostizierten Verletzungsmusters oder einer Einschränkung der Gruppenvergleichbarkeit führen könnte, bleibt unklar. Des Weiteren sollte untersucht werden, ob im Rahmen der Schockraumdiagnostik bei jüngeren Patienten durch ein aus strahlenhygienischen Aspekten zurückhaltendes Verhalten mehr relevante Diagnosen übersehen werden. Eine Studie, die Ergebnisse aus einer Datenbank untersuchte, konnte feststellen, dass eine Thorax-CT-Untersuchung bei Kindern zur Diagnosestellung einer aortalen Verletzung zu einem deutlich höheren Risiko einer kanzerogenen Entwicklung führt, als eine Verletzung aufzudecken [4].

Für die Diagnostik von Lungenkontusionen, die im Kindesalter eine hohe Inzidenz aufweisen [18], gibt es Studien, die Prädiktionsfaktoren zusammenfassen, welche die Wahrscheinlichkeit von Lungenkontusionen voraussagen sollen [10].

Auch andere Studien befassen sich mit der Etablierung von Richtlinien und Behandlungsmaßstäben bei pädiatrischen Patienten und sprechen sich insgesamt zurückhaltend gegenüber radiologischen Interventionen aus [1]. Andere Studien wiederum berichten von übersehenen Verletzungen bei jüngeren Patienten, v. a. im Bereich der oberen Extremität [16]. Insgesamt gibt es hierzu aktuell nur eine schwache Studienlage mit kontroversen Ansichten aufgrund von abweichenden diagnostischen Standards bei pädiatrischen Traumapatienten [1, 7, 17]. Es soll jedoch betont werden, dass bei allen Patienten, besonders aber bei jüngeren Patienten, die Indikationen für eine Ganzkörper- oder Bereich-CT-Diagnostik ganz gezielt und individuell in Abhängigkeit vom klinischen Zustand und Befund getroffen werden sollten.

Wir konnten in unserer Studie altersabhängige Unterschiede in Verletzungsmustern von Patienten aufdecken, die als Beifahrer oder Fahrer eines Motorrads in einen Unfall involviert waren. Dabei zeigte sich, dass bei jüngeren Patienten Verletzungen im Bereich der unteren Extremität am häufigsten vorkommen. Thorax-, abdominelle‑, Wirbelsäulen- und Beckenverletzungen hingegen weisen insgesamt eine niedrige Inzidenz auf.

### Limitationen

Durch den verwendeten Datensatz des Traumaregister DGU® ergeben sich von vornherein mehrere methodische Limitationen. Es handelt sich um eine retrospektive Analyse. Zudem sollte beachtet werden, dass viele Einflussfaktoren eine nicht zu unterschätzende Rolle bei der Bewertung der beschriebenen Ergebnisse spielen. So unterliegt z. B. das Outcome des Patienten einer Vielzahl von Faktoren (z. B. Erfahrung des Rettungsdienstpersonals vor Ort, Uhrzeit und Ort des Traumas, Rettungsmittel, versorgende Einrichtung, Patientenfaktoren), die nur unzureichend in ihrer Gesamtheit erfasst werden können. Eine Differenzierung dieser Faktoren erlaubt diese Studie nicht. Des Weiteren weisen die jeweiligen Gruppen relativ inhomogene Gruppenstärken auf, womit sich Auswirkungen auf die Ergebnisse nicht ausschließen lassen. Hier sollte auch erwähnt werden, dass die beiden jüngeren Altersgruppen weniger Ganzkörper-CT-Untersuchungen erhielten und dies ggf. zu einer Verfälschung des Verletzungsmusters sowie zu einer Einschränkung der Gruppenvergleichbarkeit führen könnte. Zuletzt bleibt festzuhalten, dass präklinisch verstorbene Patienten nicht im TraumaRegister DGU® erfasst werden.

## Fazit für die Praxis

Diese Studie konnte anhand eines großen Kollektivs (*n* Zielgruppe = 5136) zeigen, dass

- bei Jugendlichen (16 bis 17 Jahre) Verletzungen der unteren Extremität das häufigste Verletzungsmuster darstellen,
- Kinder (4 bis 15 Jahre) häufiger ein Schädel-Hirn-Trauma erleiden, jedoch im Vergleich zu höheren Altersstufen ein besseres Outcome aufweisen,
- abdominelle Verletzungen sowie Wirbelsäulen- und Beckenverletzungen bei jüngeren Altersgruppen eine niedrigere Inzidenz aufweisen,
- Rippenverletzungen im direkten Vergleich zu Lungenkontusionen bei Kindern insgesamt selten auftreten, da Kinder aufgrund der noch ausstehenden Verknöcherung einen weniger rigiden Thorax aufweisen,
- Kinder seltener einer Ganzkörper-CT-Diagnostik zugeführt werden, die gezielte CT-Diagnostik in Abhängigkeit vom klinischen Befund im Kindesalter jedoch entsprechend eine große Relevanz aufweist.
